# Disease-Associated Shifts in Minor T Cell Subpopulations Define Distinct Immunopathology in HBV vs. HCV Infection

**DOI:** 10.3390/ijms26167761

**Published:** 2025-08-11

**Authors:** Zoia R. Korobova, Natalia A. Arsentieva, Anastasia A. Butenko, Oleg K. Batsunov, Natalia E. Lyubimova, Yulia V. Ostankova, Ekaterina V. Anufrieva, Sergey A. Maslov, Konstantin V. Kozlov, Dmitrii L. Sulima, Oksana Yu. Rishnyak, Areg A. Totolian

**Affiliations:** 1 Saint Petersburg Pasteur Institute, Mira St. 14, 197101 St. Petersburg, Russia; arsentieva_n.a@bk.ru (N.A.A.);; 2 Department of Immunology, Pavlov First State Medical University of St. Petersburg, L’va Tolstogo St. 6-8, 197022 St. Petersburg, Russia; 3 Military Medical Academy Named After S.M. Kirov, Department of Infectious Diseases, Academika Lebedeva St., 6Zh, 194044 St. Petersburg, Russia; 4 Medical Clinic Exclusive, Korablestroiteley St. 33, Building 2, 199058 St. Petersburg, Russia; uncledimamed@mail.ru; 5 Vsevolozhsk Clinical Hospital, Koltushskoe Shosse, 20, 188643 Vsevolozhsk, Russia

**Keywords:** T cells, viral hepatitis, HBV, HCV, naïve T cell, central memory cells, effector memory cells, terminally differentiated effector memory cells, polarized T cell

## Abstract

Hepatic viruses, such as hepatitis B and C (HBV and HCV), evade immune defenses and drive liver cirrhosis and cancer. They remain a major global health burden, requiring deeper research into immune responses; specifically, adaptive immunity. This study aims to analyze T cellular subsets in chronic HBV and HCV infection and investigate their potential role in the immunopathogenesis of these conditions. Methods: For our study, we collected 123 blood samples taken from patients infected with HCV (*n* = 36) and HBV (*n* = 34) and healthy volunteers (*n* = 53). With the use of flow cytometry, we assessed levels of CD4+ and CD8+ minor T cell subpopulations (naïve, central, and effector memory cells (CM and EM), terminally differentiated EM (TEMRA), Th1, Th2, Th17, Tfh, Tc1, Tc2, Tc17, Tc17.1). Results: Despite similar total CD4+ T cell frequencies across chronic HCV, HBV, and healthy groups, patients with hepatitis showed elevated TEMRA, EM, and CM subsets alongside depleted naïve Th cells and specific CM subpopulations compared to controls. Patients with chronic HCV and HBV showed elevated CD8+ T cell frequencies versus controls, with disease-specific shifts: reduced EM CTLs but increased TEMRA CTLs, Tc1/Tc17.1 depletion (notably Tc17.1 in HCV), and higher Tc2 levels. Conclusions: Viral clearance in HBV and HCV requires a delicate balance between immunity and viral activity. Despite similar T cell frequencies (CD3+/CD4+/CD8+), minor subsets revealed distinct patterns differentiating HCV, HBV, and healthy controls.

## 1. Introduction

Hepatitis is an inflammatory condition of the liver. It is generally caused either by infectious agents, primarily viruses, or noninfectious factors (e.g., alcohol or toxic substances). Hepatitis B virus (HBV) and hepatitis C (HCV) virus pose the greatest public health threat due to their potential to cause chronic infection, leading to progressive liver fibrosis, cirrhosis, and hepatocellular carcinoma. WHO epidemiological data (covering 187 countries) highlight viral hepatitis as a critical health challenge of the current decade. In 2022 alone, chronic hepatitis B and C were responsible for 1.3 million deaths worldwide, equating to approximately 3500 lives lost per day. As of recent estimates, 254 million people are living with chronic hepatitis B, while 50 million are affected by hepatitis C. Furthermore, around 6000 new infections occur daily [1].

Viral hepatitis pathogens, particularly HBV and HCV, have developed complex mechanisms to evade and manipulate the host immune system, driving chronic liver disease. HBV, often called a “stealth virus,” initially avoids detection by minimizing innate immune activation, but recent studies reveal it actively suppresses pathways like the cGAS-STING-IFN axis through viral microRNAs (e.g., HBV-miR-3), which downregulate cGAS expression and impair interferon production [2]. HCV proteins can block the production of IFN-α/β, crucial antiviral cytokines, by interfering with signaling pathways like the RIG-I pathway. HCV core protein can suppress cytotoxic T lymphocyte (CTL) responses and cytokine production [3].

Research into T cell immune responses is crucial for understanding chronic HBV and HCV infection. Both infections feature dysfunctional T cells, especially CTLs and CD4+ helper T cells. This impairment is a key reason the immune system fails to clear the viruses.

Affected T cells show signs of exhaustion, including reduced release of critical immune chemicals (IFNγ, TNFα, IL-2) and weakened ability to kill infected cells. Furthermore, defective CD4+ helper T cell function undermines support for both CD8+ T cells and antibody-producing B cells. This includes impaired T follicular helper (Tfh) cell activity, which disrupts the germinal center reactions essential for effective antibody development [4,5].

Based on the critical role of T cell dysfunction and exhaustion in chronic HBV/HCV persistence, evaluating minor T cell subpopulations—particularly those involved in memory and effector differentiation—becomes essential for assessing immune potential and disease control [6,7]. Researching these subpopulations may help answer the following questions: what leads to the infectious process becoming chronic in the first place, and how does immune imbalance affect disease progression?

## 2. Results

**Figure 1:**
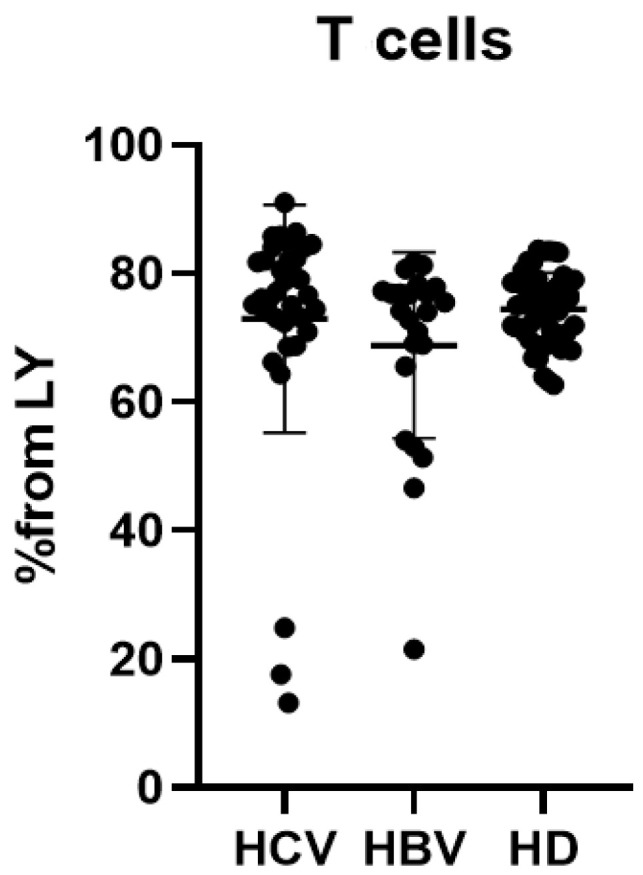
Percentage of CD3+ T lymphocytes from all CD45+ cells for patients with chronic HCV infection (HCV, *n* = 36) and chronic HBV infection (HBV, *n* = 34) and healthy donors (HD, *n* = 53).

In the initial phase of this study, we measured the proportion of CD3+ T cells within the total lymphocyte (CD45+) fraction across cohorts with chronic HCV infection, chronic HBV infection, and healthy donors (Figure 1).

While overall CD3+ T cell frequencies showed no significant differences between groups, we hypothesized that disease-specific immune polarization—influenced by infection type and severity—might manifest in T cell subset distribution. We therefore characterized major CD4+ and CD8+ T cell populations, followed by comprehensive profiling of minor functional subsets within these compartments.

Absolute numbers of CD3+ T cells demonstrated a decrease in the HBV cohort (Table 1).

**Figure 2:**
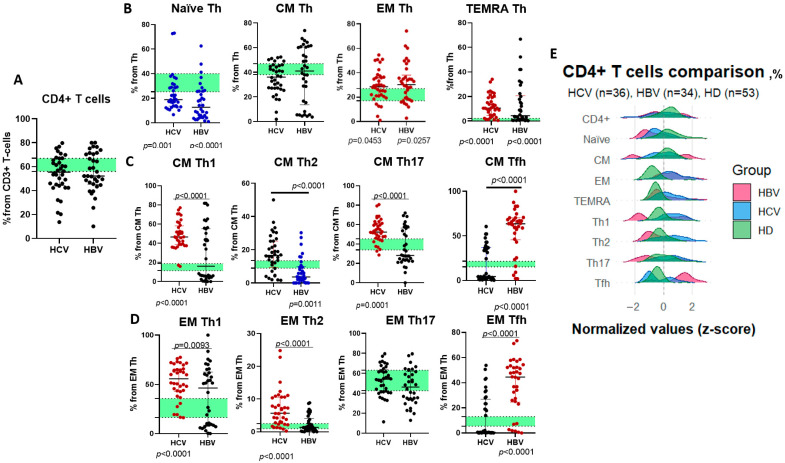
(**A**)—CD4+ lymphocyte percentages among CD3+ T cells in patients with chronic HBV (*n* = 34, HBV) and chronic HCV (*n* = 36, HCV); (**B**)—Naïve Th cells, central memory (CM) Th cells, effector memory (EM) cells, and terminally differentiated cells (TEMRA) in patients with chronic HBV (*n* = 34, HBV) and chronic HCV (*n* = 36, HCV); (**C**)—Minor subsets of CM cells—Th1, 2, 17, follicular T helpers (Tfh) in patients with chronic HBV (*n* = 34, HBV) and chronic HCV (*n* = 36, HCV). (**D**)—Minor subsets of EM cells—Th1, 2, 17, follicular T helpers (Tfh) in patients with chronic HBV (*n* = 34, HBV) and chronic HCV (*n* = 36, HCV). (**E**)—Ridgeplot for CD4+ T cell subsets in patients with chronic HBV (*n* = 34, red plot) and chronic HCV (*n* = 36, blue plot) and healthy donors (*n* = 53, green plot). (**A**–**D**)—Lines represent medians and Q25–Q75 intervals. Green stripe represents Q25–Q75 in healthy donors. Blue dots represent lower cell percentages when compared to healthy donors (*p* < 0.05), red dots represent higher cell percentages when compared to healthy donors (*p* < 0.05). *p*-values for comparison of each cohort with healthy controls are shown below the X axis. Ridgeline plot (E) displays density distributions of normalized (z-score) frequencies for CD4+, naïve, CM, EM, TEMRA, Th1, Th2, Th17, and Tfh subsets.

As total CD4+ T helper cell frequencies did not differ significantly between chronic HCV, HBV, and healthy cohorts (Figure 2A), a redistribution was observed across memory subsets. Terminally differentiated effector (TEMRA), effector memory (EM), and select central memory (CM) subsets were significantly higher in both hepatitis groups vs. controls (Figure 2B). At the same time, naïve T helpers were depleted, whereas changes in CM/EM Th1-Th17 subsets were seen (Figure 2C,D). Disease-specific polarization patterns were seen—patients with HCV showed elevated CM Th1/Th17 cells and EM Th1/Th2 subsets, whereas chronic HBV infection featured expanded T follicular helper (Tfh) cells (Figure 2E).

In this study, we analyzed not only the relative distribution (in percentages) among T helper populations and minor subsets but also their absolute counts. While the differences between these two analytical approaches were minor, it is important to emphasize that the total CD4+ T cell counts were significantly lower in HCV and HBV infections than in controls. Additionally, we observed a notable reduction in central memory (CM) Th lymphocytes and Th17 cells within the EM fraction (Table 2).

**Figure 3:**
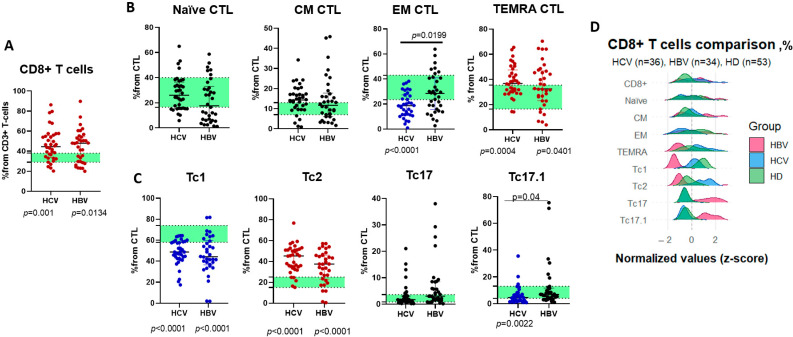
(**A**)—CD8+ lymphocyte percentages among CD3+ T cells in patients with chronic HBV (*n* = 34, HBV) and chronic HCV (*n* = 36, HCV). (**B**)—Naïve, central memory (CM), effector memory (EM), and terminally differentiated (TEMRA) CTL percentages among CD8+ cells in patients with chronic HBV (*n* = 34, HBV) and chronic HCV (*n* = 36, HCV). (**C**)—Cytokine-producing CTL (Tc) Tc1, Tc2, Tc17, and Tc17.1 percentages among CD8+ cells in patients with chronic HBV (*n* = 34, HBV) and chronic HCV (*n* = 36, HCV). (**D**)—Ridgeplot for CD8+ cytotoxic (CTL) T cell subsets in patients with chronic HBV (*n* = 34, red plot), chronic HCV (*n* = 36, blue plot), and healthy donors (*n* = 53, green plot). (**A**–**C**)—Lines represent medians and Q25–Q75 intervals. Green stripe represents Q25–Q75 in healthy donors. Blue dots represent lower cell percentages when compared to healthy donors (*p* < 0.05), red dots represent higher cell percentages when compared to healthy donors (*p* < 0.05). *p*-values for comparison of each cohort with healthy controls are shown below the X axis. Ridgeline plot (**D**) displays density distributions of normalized (z-score) frequencies for CD8+, naïve, CM, EM, TEMRA, Tc1, Tc2, Tc17, and Tc17.1 subsets.

Analysis of CD8+ T cell distribution revealed significantly elevated frequencies in both chronic HCV and HBV cohorts compared to healthy controls (Figure 3A). Normalization and subsequent ridgeplotting identified distinct polarization patterns—TEMRA, Tc1, and Tc2 subsets were consistently higher in patients with HCV (Figure 3B,C). Further characterization of minor populations demonstrated a disease-associated shift, with reduced effector memory (EM) CTLs in HCV/HBV groups vs. controls, contrasting with elevated TEMRA CTLs (Figure 3C). Subset profiling revealed significant changes in cytotoxic subsets—Tc1 and Tc17.1 cells were depleted (Tc17.1 most prominently in HCV), while Tc2 cells were higher in both hepatitis groups (Figure 3D).

While the relative distribution of CD8+ T cell subsets showed significant variation across groups, we also observed a reduction in absolute counts in HBV infection compared to controls (Table 3). Notably, individuals who were HBV-infected exhibited decreased absolute numbers of naïve cytotoxic T lymphocytes (CTLs), suggesting potential implications for immune memory formation or antiviral responses.

## 3. Discussion

### 3.1. Changes in T Cellular Responses Associated with HCV Infection

In HCV infection, key factors for successful clearance of the virus are high levels of circulating CD8+ CTLs, high IFN production, and post-clearance CTL fluctuation, suggesting ongoing low-level antigen exposure. Acute HCV is resolved when the cytotoxic activity of T cells outbalances the viral mechanisms of immune evasion [8,9]. Thus, a mere 30% of patients infected with HCV are able to clear the infection, as the majority progress to chronic infection [10]. While recovery from HCV does not provide sterilizing immunity, it still provides protection. The acquired immunity may not prevent reinfection but significantly reduces the risk of chronicity compared to a primary infection [11]. One important role in HCV clearance belongs to follicular T helpers, as they coordinate humoral responses to this infection [12]; however, early introduction of these cells to immune responses may contribute to viral expansion [13]. Our study demonstrated that these cells are depleted in chronic infection.

In chronic HCV, several specific changes are usually seen. As our results demonstrate, memory cells prevail among both CD4+ cells and CD8+ cells. Naïve cells, specifically Th cells, are lowered, possibly due to the chronic nature of the infectious process in the studied cohort. A hallmark of chronic HCV is sustained T cell exhaustion. While our study did not evaluate PD-1 or other exhaustion markers, we quantified highly differentiated TEMRA Th cells and CTLs, which often express PD-1 yet retain potent cytotoxic activity [14]. Both murine models and human HCV studies further indicate that exhausted T cells are heterogenous—some cells retain proliferative capacity while others are terminally exhausted [15]. A critical insight into T cell memory in chronic HCV infection is that virus-specific CD8+ T cells undergo persistent phenotypic and functional reprogramming, which remains present even after successful recovery. As demonstrated by Hensel et al. (2021) [16], these cells retain a distinct “molecular scar”—reflecting sustained epigenetic and transcriptional alterations—long after antigen clearance. This aligns with observations by Hofmann et al. (2018) [17] that chronic HCV drives the development of “memory-like” CD8+ T cells, which differ functionally and phenotypically from classic memory T cells generated during acute/resolved infections. While these cells persist post-cure, their ability to provide effective antiviral responses (e.g., cytotoxicity, cytokine production) upon re-exposure to HCV is poorly defined. It is unclear whether such “scarred” or “memory-like” CD8+ T cells can effectively prevent de novo infection or control viral replication during reinfection.

Chronic HCV infection within our study demonstrated elevation across all major T helper (Th) subsets—Th1, Th2, and Th17. A pronounced Th1 response, characterized by dominant IFN-γ and TNF-α production, is a well-established feature of HCV immunity [18]. Interestingly, when exposed to the HCV F protein, peripheral blood mononuclear cells (PBMCs) from infected patients adopt distinctive Th2-polarized responses (e.g., production of IL-4, IL-5) [19]. Th17 activation correlates with poorer clinical outcomes—patients exhibiting lower Th17 activity respond better to antiviral therapy [20]. This response is actively subverted by viral immune evasion strategies. As demonstrated by Rowan et al., HCV suppresses virus-specific Th1 and Th17 cells via the NS4 protein, which triggers immunosuppressive IL-10 and TGF-β production from innate immune cells [21]. IL-17 and Th17 cells are important tissue repair agents in liver tissue damage; they are present not only in the peripheral blood but also in liver tissue [22].

Since changes in Th cells were not specific—we noted elevation in all subsets—we addressed changes in cytokine-producing T cytotoxic cells, counterparts of T helpers. Our findings revealed a significant depletion of Tc1 and Tc17 cells, suggesting that cytotoxic T cell (CTL) polarization may offer more precise insights into immune polarization dynamics than helper T cell subsets. This is likely due to the progressive dysfunction of helper T cells as infection advances, as evidenced by prior research [23]. We also noted an interesting diversity of immune responses regarding central memory and effector memory cells. While central memory cells reside in lymphoid tissues, effector memory cells migrate with the peripheral blood stream [24]. While helper central memory cells were lowered and effector memory cells increased, the immune landscape was the complete opposite for cytotoxic lymphocytes.

Cytotoxic Tc1 and Tc17 cells are the CD8+ counterparts of Th1 and Th17 cells, mirroring their cytokine profiles—Tc1 produces IFN-γ, while Tc17 secretes IL-17—but with direct cytotoxic effector functions. The observed depletion of these subsets underscores their critical role in immune regulation and highlights their potential as more reliable indicators of shifting immune responses than their helper cell analogs, particularly in chronic or progressive infections where helper T cell dysfunction becomes more pronounced.

In our prior study on chronic HCV infection, cytokine profiling revealed a similar immunological pattern. Th1/Tc1-associated cytokines (IFN-γ, IL-2) and Th2/Tc2-related mediators (IL-4, IL-10, IL-13) were either elevated in patients with HCV or showed no significant difference compared to healthy donors. Interestingly, despite reduced Tc17 cell frequencies and expanded Th17 populations, all IL-17 isoforms were markedly elevated in HCV infection. This finding highlights the predominance of Th17 cells over Tc17 subsets in cytokine production and the general direction of immune polarization during chronic HCV infection [25].

### 3.2. Changes in T Cellular Responses Associated with HBV Infection

For the successful clearance of HBV infection, T cellular responses need to be broad and multispecific (CD8+/CD4+) against multiple HBV proteins (core, envelope, polymerase). Patients with chronic infection demonstrate minimal or even absent T cellular responses [26]. In our study, cellular responses in HBV infection were present, though percentages of the CD8+ effector memory cells and Tc1 cells crucial for elimination of the virus were lowered. At the same time, similar to chronic HCV infection, the naïve fraction of cells was also depleted.

In patients with controlled HBV infection, activation of the adaptive immune system is held under control from the stance of both T and B cells, which is indispensable for clearing the majority of infected hepatocytes and maintaining control of the virus [27]. Recent findings indicate that the dysfunction seen in HBV-specific CD8+ T cells does not always conform to the typical exhaustion profile. While exhausted cells are found in low numbers in patients with chronic HBV, they display a diverse range of inhibitory receptor expression and effector functions. For example, the expression levels of PD1, TIM3, CTLA4, and CD160 vary among these cells, but these markers do not reliably reflect the extent of their functional impairment. This variability implies that there are additional, non-traditional mechanisms influencing the responses of HBV-specific CD8+ T cells during chronic infection [28]. Looking into cellular subsets may provide insights into the possibility of recovery [29].

In cases of chronic hepatitis B virus infection, extended exposure to HBV or HBsAg/hepatitis B e antigen (HBeAg) can hinder antigen-presenting cells (APCs) from delivering sufficient signals 1 and 2 necessary for T cell activation. This issue, along with a deficiency of activating cytokines and the presence of inhibitory molecules, may ultimately result in the dysfunction of HBV-specific CD8 T cells [30].

Our study demonstrated a certain depletion in effector memory T cells and Tc1 cells. This finding presents a certain point of interest, as these cells are one of the major effector players in anti-HBV responses. When stimulated with HbsAg, Tc1 cells demonstrated induced cytotoxicity [31]. Yet, in patients with chronic HBV infection, Tc1 cells are depleted—a finding potentially explaining how the disease course turns from acute to chronic.

Another interesting finding regards Th2-mediated immune responses. In HBV infection, we noted depletion in Th2 cells and a subsequent increase in T follicular helpers. In a review article by Vanwollengham, humoral responses, which are often mediated via Th2 and follicular helper cells, are named as important factors for anti-HBV immunity [32].

We also noted an absolute depletion in CD8+ T cells in chronic HBV infection. Recent studies highlight that HBV as a virus demonstrates certain lymphotropism with regards to CD8+ T cells [33].

Such findings suggest weakened responses in major players for anti-HBV immunity; yet, as compensation, we see an elevation in other immune chain links—TEMRA and Tc2 cells. The first subset represents final effector antigen-specific cells, and Tc2 acts as a collateral source for the Th2 cytokines that the Th2 cells are lacking.

In the present study, we observed a reduced proportion of Th17 (CD4+) cells alongside an increased frequency of Tc17 (CD8+) cells in individuals who were HBV-infected. Since Th17 cells are considered the primary producers of IL-17A, their depletion may explain the overall reduction in this cytokine despite the relative expansion of Tc17 cells. This suggests that Tc17 cells, while present in greater numbers, may contribute less significantly to systemic IL-17A-mediated immune polarization than their CD4+ counterparts [34]. Similarly, we found significant depletion of both Th1/Tc1 and Th2/Tc2 populations in HBV infection. However, the associated cytokine profiles revealed distinct dynamics—while IFN-γ and IL-2 (linked to Th1 polarization) were reduced only in severe fibrosis, Th2-associated cytokines (IL-4, IL-10, IL-13) were consistently low regardless of disease stage. This dissociation between cellular frequencies and cytokine levels implies that functional exhaustion or altered secretory abilities exist.

### 3.3. Disease-Specific Shifts in Immune Cell Populations: HBV vs. HCV

**Figure 4:**
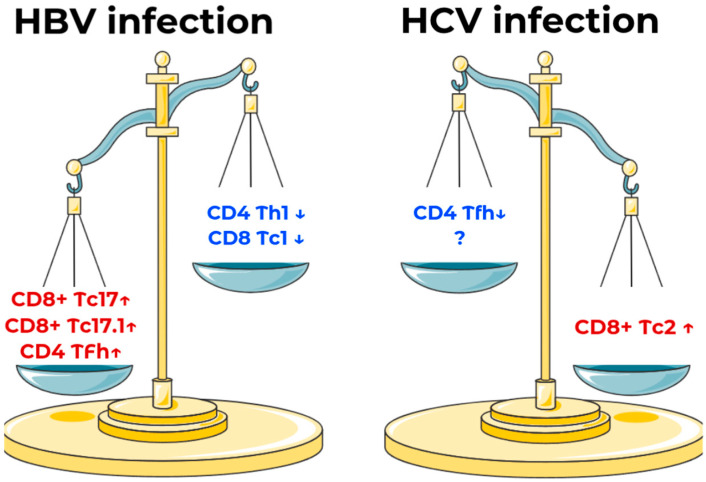
Disease-specific changes in immune cells based on results of statistical analysis and data normalization for HBV and HCV infection. ↓—redistribution of normalized cells percentages is lower than the global mean across all three groups; ↑—redistribution of normalized cells percentages is higher than the global mean across all three groups. As seen on the illustration, immune polarization for HBV infection is skewed towards cytokine-producing cytotoxic T lymphocytes (Tc) 17 and 17.1, follicular T helper cells, whilst HCV infection is characterized by a dominance of CD8+ Tc2 cells and a lack of follicular helper cells. Dominance of certain subpopulations allows two infections to be distinguished from the immunological stance.

While the major T cell populations (CD3+, CD4+, CD8+) did not differ significantly across groups, a detailed investigation of minor T cell subpopulations revealed profoundly different immunological patterns distinguishing HCV, HBV, and healthy donors. Moreover, while the two infections (HBV and HCV) are quite similar in terms of clinical presentation, disease-specific immunity shifts differ (Figure 4).

Our study shows clear differences in immune responses between HBV and HCV infections. In HBV, we saw more Tc17 and Tc17.1 cells, which matches previous findings about their role in chronic infections [35]. Unlike typical viral infections, HBV showed fewer Tc1 cells—these are the powerful virus-fighting cells that somehow fail to clear HBV [36]. Helper Th1 cells, which normally support this fight, were also reduced. The increase in T follicular helper (Tfh) cells in HBV suggests problems with antibody production, as seen in mouse studies [37].

HCV showed a different pattern, with more CD8+ Tc2 cells. These cells are usually discussed in terms of allergic inflammation [36], so their role in HCV requires more research. We also noticed slightly fewer Tfh cells in HCV than in HBV. These findings show how differently these two viruses affect immunity, and we welcome further studies on other cell types we might have missed.

### 3.4. Study Limitations and Future Perspective

This study has several limitations that should be acknowledged. First, we did not assess exhaustion markers (e.g., PD-1 and related molecules), which are critical for evaluating cellular senescence and life-cycle stages, potentially limiting our understanding of T cell functionality in the observed context. Additionally, cytokine levels were not measured, restricting our ability to analyze intercellular communication and immune modulation—an important aspect that will be addressed in future research. While this study provides insights into the redistribution of T cell subsets, future work should integrate transcriptional profiling to refine lineage-specific characterization. The inclusion of key regulators such as TBX21 (TH1), GATA3 (TH2), and BCL6 (TFH) would strengthen the biological interpretation of subset dynamics, particularly in distinguishing phenotypically overlapping EM populations. Another limitation is the reliance on peripheral blood samples rather than hepatic tissue biopsies. While blood-based analyses provide valuable data on systemic responses, tissue-resident immune cells may more accurately reflect localized immune responses in hepatitis infections. Future studies on biopsy material could offer deeper insights into compartment-specific immunity.

## 4. Materials and Methods

### 4.1. Studied Cohorts

The research was conducted across multiple medical institutions in Saint Petersburg and the Leningrad Region, including the Saint Petersburg Pasteur Institute, Military Medical Academy named after S.M. Kirov, Medical Clinic EXCLUSIVE, and Vsevolozhsk Clinical Hospital. Ethical approval was granted by the Saint Petersburg Pasteur Institute’s Ethical Committee (Protocol # 161/a, 19 January 2023; Protocol # 206, 24 September 2024), and all participants provided written informed consent. For our study, we collected 123 blood samples taken from patients infected with HCV (*n* = 36) and HBV (*n* = 34) and from healthy volunteers (*n* = 53). Patients’ baseline characteristics are presented in Table 4.

Diagnoses were confirmed through clinical evaluation, imaging (ultrasound and elastometry), and laboratory testing conducted by specialists at collaborating medical institutions. HCV infection was verified via anti-HCV antibodies and HCV RNA detection in peripheral blood. HBV infection was assessed using standard serological markers: HBsAg, anti-HBs IgG, anti-HBc IgG (total/core), HBeAg, anti-HBe IgG, and HBV DNA quantification.

The median age for the HCV cohort was 52 years (Q25–39; Q75–67) and 48 years (36; 66) for the HBV cohort. In the HCV cohort, 41.6% (*n* = 13) of patients were male and 58.4% (*n* = 23) were female. In the HBV cohort, 29,4% were male (*n* = 10) and 70,6% (*n* = 24) were female.

The healthy donor cohort consisted of 53 healthy individuals (23 male and 30 female). The median age was 40 (34; 49) years.

### 4.2. Sample Collection

Blood samples were collected before treatment initiation. Peripheral blood samples (5 mL) were obtained from each patient using VACUETTE^®^ K3 EDTA tubes (Griener Bio-One, Kremsmünster, Austria). T cell immunophenotyping was conducted within 6 h of blood collection.

### 4.3. T Cell Immunophenotyping by Flow Cytometry

To identify and determine T lymphocytes (CD3+), cytotoxic (CD3+CD8+) T lymphocyte subsets, and helper cells (CD3+CD4+), the following combination of antibodies was used: CD3-FITC, CD8-PE, CD45-PerCP, and CD4-APC (Becton Dickinson, Franklin Lakes, NJ, USA).

For the immunophenotyping of the maturation stages and “polarized” CD4+ and CD8+ T cell subsets, the following combination of antibodies was employed: CD45RA-FITC, CD62L-PE, CD3-APC-AF750, CD4-Pacific Blue or CD8- Pacific Blue, CXCR5-PerCP/Cy5.5, CCR6-PE/Cy7, CXCR3-APC, and CCR4-Brilliant Violet 510 (CD45RA and CD62L were manufactured by Beckman Coulter, Indianapolis, IN, USA, with the remaining other antibodies purchased from BioLegend, Inc., San Diego, CA, USA).

Earlier, we described the gating strategy for T cell subset maturation stages and the major “polarized” T cell subsets [38,39].

A total of 200 μL of whole blood was stained with an antibody cocktail (optimized per the manufacturer guidelines) in the dark at room temperature for 15 min. Erythrocyte lysis was performed using 2 mL of VersaLyse Lysing Solution (Beckman Coulter, Indianapolis, IN, USA), supplemented with 50 μL of IOTest 3 Fixative Solution (15 min incubation, dark, RT). The cells were washed with PBS, centrifuged (330× *g*, 7 min), and resuspended in 500 μL of PBS containing 2% neutral formalin (Sigma-Aldrich, Burlington, MA, USA). Samples were analyzed on an Acea Novocyte flow cytometer (Agilent Technologies, Santa Clara, CA, USA).

### 4.4. Statistical Analysis

Between-group differences were assessed using the Kruskal–Wallis test for comparisons across >2 groups, with Dunn’s post-hoc test for pairwise comparisons where applicable. For comparisons involving two groups, the Mann–Whitney U test was employed. These comparisons and visualizations were made with GraphPad Prism 8 (Dotmatics, Boston, MA, USA). Subpopulation frequencies were normalized using z-score transformation within each subset with R Studio (2025.05.1 build 513). Visualization included ridgeline plots (ggridges package v0.5.6), displaying density distributions of normalized values for each subpopulation with direct HBV vs. HCV vs. HD group comparisons. All plots used consistent parameters (x-axis limits: z-score ± 3; dimensions: 6 × 10 inches). For visualization, we used the tidyverse (v2.0.0) and ggplot2 (v3.5.1) packages.

## 5. Conclusions

While major T cell populations (CD3+, CD4+, CD8+) showed comparable frequencies across chronic HBV, HCV, and healthy groups, our detailed analysis of minor subsets uncovered fundamentally distinct immunological signatures distinguishing these conditions. Despite clinical similarities between HBV and HCV, the diseases exhibited opposing immune polarizations—HBV was characterized by expansions of cytokine-producing Tc17/Tc17.1 cells and follicular T helpers (Tfh), whereas HCV demonstrated dominance of CD8+ Tc2 cells with relative Tfh depletion. Both infections shared features of immune dysregulation—HBV with its deficit in virus-clearing Tc1/Th1 cells, and HCV with its paradoxical Tc2 skewing, typically associated with allergic inflammation.

These disease-specific shifts likely contribute to viral persistence and liver pathology, highlighting the need to investigate how these subsets mechanistically influence disease progression. Our findings highlight that adaptive immunity’s failure in hepatitis is hidden not in major T cell population numbers but in precise subset imbalances. Future studies should explore therapeutic modulation of these subsets and address unanswered questions about their functional roles in viral clearance and liver damage.

## Figures and Tables

**Table 1:** Comparison of CD3+ T cell percentages and absolute counts (×10^9^/L) in HCV-infected (*n* = 36), HBV-infected (*n* = 34), and healthy donor (HD, *n* = 53) groups.

		HCV (1)	HBV (2)	HD (3)	*p*-Value (Kruskal–Wallis)
T cell	%	76.4 (72.54; 82.23)	74.22 (65.58; 77.3)	75.09 (70.42; 78.71)	ns
	abs	1.64 (1.25; 1.86)	0.98 (0.69; 1.61)	1.25 (1.06; 1.60)	*p* (1–2) = 0.0103

Note: Data are presented as median (IQR). Kruskal–Wallis testing was used for analysis. ns—non significant differences (*p* > 0.05).

**Table 2:** Comparison of CD3+CD4+ T cell major and minor population percentages and absolute counts (×10^9^/L) in HCV-infected (*n* = 36), HBV-infected (*n* = 34), and healthy donor (HD, *n* = 53) groups.

		HCV (1)	HBV (2)	HD (3)	*p*-Value (Kruskal–Wallis)
CD4+ T cell	%	55.46 (44.27; 66.55)	52.23 (45.28; 70.79)	62.94 (56.2; 66.82)	ns
	abs	0.60 (0.25; 0.99)	0.31 (0.10; 0.59)	0.77 (0.67; 1.07)	*p* (1–3) = 0.0338 *p* (2–3) < 0.0001
Naïve Th	%	18.86 (13.71; 29.18)	12.68 (4.465; 25.83)	31.3 (25.3; 39.8)	*p* (1–3) = 0.001 *p* (2–3) < 0.0001
	abs	0.12 (0.04; 0.20)	0.0334 (0.01; 0.08)	0.22 (0.19; 0.33)	*p* (1–2) = 0.0327 *p* (1–3) = 0.0016 *p* (2–3) < 0.0001
CM Th	%	36.13 (27.1; 45.24)	41.06 (13.6; 60.52)	41.55 (37.66; 47.33)	ns
	abs	0.17 (0.08; 0.33)	0.12 (0.06; 0.37)	0.33 (0.27; 0.47)	*p* (1–3) = 0.0016 *p* (2–3) = 0.0012
EM Th	%	28.59 (21.0; 36.32)	30.33 (18.42; 38.06)	22.94 (17.28; 27.45)	*p* (1–3) = 0.0453 *p* (2–3) = 0.0257
	abs	0.19 (0.05; 0.20)	0.07 (0.05; 0.17)	0.18 (0.14; 0.23)	*p* (1–3) = 0.0212 *p* (2–3) = 0.0003
TEMRA Th	%	10.57 (4.67; 19.23)	4.505 (1.09; 20.68)	0.68 (0.27; 2.58)	*p* (1–3) < 0.0001 *p* (2–3) < 0.0001
	abs	0.03 (0.01; 0.09)	0.01 (0.01; 0.03)	0.001 (0.02; 0.02)	*p* (1–2) = 0.0151 *p* (1–3) < 0.0001
CM Th1	%	46.67 (38.79; 58.21)	14.81 (11.4; 18.8)	14.81 (11.4; 18.8)	*p* (1–2) < 0.0001 *p* (1–3) < 0.0001
	abs	0.07 (0.03; 0.15)	0.01 (0.01; 0.02)	0.05 (0.03; 0.06)	*p* (1–2) < 0.0001 *p* (2–3) < 0.0001
CM Th2	%	16.61 (9.28; 25.22)	10.72 (8.92; 13.4)	10.72 (8.92; 13.4)	*p* (1–2) < 0.0001 *p* (2–3) = 0.0011
	abs	0.02 (0.01; 0.07)	0.02 (0.01; 0.02)	0.04 (0.03; 0.05)	*p* (1–2) = 0.0005 *p* (2–3) < 0.0001
CM Th17	%	52.32 (45.42; 61.1)	39.23 (33.93; 45.26)	39.23 (33.93; 45.26)	*p* (1–2) < 0.0001 *p* (1–3) = 0.0001
	abs	0.08 (0.04; 0.18)	0.03 (0.01; 0.09)	0.12 (0.09; 0.17)	*p* (1–2) = 0.0343 *p* (2–3) < 0.0001
CM Tfh	%	4.38 (2.63; 36.77)	17.87 (15.23; 21.63)	17.87 (15.23; 21.63)	*p* (1–2) < 0.0001 *p* (2–3) < 0.0001
	abs	0.02 (0.01; 0.07)	0.07 (0.01; 0.19)	0.06 (0.05; 0.08)	*p* (1–2) = 0.0073 *p* (1–3) = 0.0044
EM Th1	%	55.77 (42.93; 65.67)	21.7 (16.41; 35.74)	21.7 (16.41; 35.74)	*p* (1–2) = 0.0093 *p* (1–3) < 0.0001
	abs	0.04 (0.02; 0.13)	0.01 (0.01; 0.03)	0.04 (0.02; 0.06)	*p* (1–2) = 0.0002 *p* (2–3) = 0.0001
EM Th2	%	5.67 (2.43; 10.56)	1.45 (0.93; 2.47)	1.45 (0.93; 2.47)	*p* (1–2) < 0.0001 *p* (1–3) < 0.0001
	abs	0.01 (0.01; 0.02)	0.01 (0.002; 0.03)	0.01 (0.0016; 0.03)	*p* (1–2) < 0.0001 *p* (1–3) = 0.0098 *p* (2–3) = 0.0483
EM Th17	%	54.17 (41.47; 64.16)	55.45 (42.56; 63.19)	55.45 (42.56; 63.19)	ns
	abs	0.05 (0.02; 0.14)	0.03 (0.01; 0.07)	0.10 (0.07; 0.13)	*p* (2–3) = 0.0001
EM Tfh	%	0.65 (0.30; 26.88)	8.29 (5.39; 13.14)	8.29 (5.39; 13.14)	*p* (1–2) < 0.0001 *p* (2–3) < 0.0001
	abs	0.02 (0.0001; 0.03)	0.03 (0.02; 0.04)	0.02 (0.01; 0.03)	*p* (1–2) < 0.0001 *p* (1–3) = 0.0478 *p* (2–3) = 0.0495

Note: Data are presented as median (IQR). Kruskal–Wallis testing was used for analysis. ns—non significant differences (*p* > 0.05).

**Table 3:** Comparison of CD3+CD8+ T cell major and minor population percentages and absolute counts (×10^9^/L) in HCV-infected (*n* = 36), HBV-infected (*n* = 34), and healthy donor (HD, *n* = 53) groups.

		HCV (1)	HBV (2)	HD (3)	*p*-Value (Kruskal–Wallis)
CD8+ T cell	%	44.55 (33.45; 55.73)	47.93 (29.38; 54.4)	33.34 (28.94; 38.26)	*p* (1–3) = 0.001 *p* (2–3) = 0.0134
	abs	0.50 (0.27; 0.72)	0.18 (0.09; 0.34)	0.44 (0.35; 0.53)	*p* (1–2) = 0.0026 *p* (2–3) = 0.0012
Naïve CTL	%	26.06 (15.55; 36.53)	17.61 (6.093; 33.03)	29.43 (16.6; 39.61)	ns
	abs	0.10 (0.06; 0.21)	0.02 (0.01; 0.08)	0.12 (0.07; 0.15)	*p* (1–2) = 0.0011 *p* (2–3) = 0.0008
CM CTL	%	14.3 (9.88; 17.36)	11.71 (6.05; 17.79)	11.0 (6.99; 13.45)	ns
	abs	0.05 (0.02; 0.10)	0.05 (0.01; 0.06)	0.04 (0.03; 0.07)	ns
EM CTL	%	18.73 (11.56; 27.38)	28.52 (15.59; 40.92)	34.13 (23.53; 42.8)	*p* (1–2) = 0.0199 *p* (1–3) < 0.0001
	abs	0.06 (0.03; 0.11)	0.04 (0.03; 0.10)	0.15 (0.10; 0.20)	*p* (1–3) = 0.0002 *p* (2–3) = 0.0001
TEMRA CTL	%	36.8 (30.13; 47.58)	32.51 (22.95; 46.81)	25.18 (16.48; 35.55)	*p* (1–3) = 0.0004 *p* (2–3) = 0.0401
	abs	0.19 (0.10; 0.31)	0.05 (0.02; 0.14)	0.11 (0.06; 0.17)	*p* (1–2) = 0.0002 *p* (2–3) = 0.0279
Tc1	%	48.76 (42.49; 58.44)	44.29 (36.51; 59.79)	68.62 (57.78; 74.34)	*p* (1–3) < 0.0001 *p* (2–3) < 0.0001
	abs	0.24 (0.13; 0.35)	0.10 (0.04; 0.21)	0.30 (0.24; 0.37)	*p* (2–3) < 0.0001
Tc2	%	45.28 (33.94; 51.05)	37.45 (22.98; 46.06)	18.5 (14.78; 25.53)	*p* (1–3) < 0.0001 *p* (2–3) < 0.0001
	abs	0.22 (0.11; 0.35)	0.06 (0.03; 0.14)	0.09 (0.06; 0.12)	*p* (1–2) < 0.0001 *p* (1–3) = 0.0003
Tc17	%	1.685 (0.83; 3.05)	3.03 (1.523; 8.448)	1.8 (0.845; 3.665)	ns
	abs	0.01 (0.01; 0.02)	0.01 (0.01; 0.02)	0.01 (0.01; 0.02)	ns
Tc17.1	%	4.60 (2.04; 6.88)	6.89 (4.10; 11.64)	8.41 (4.00; 12.94)	*p* (1–2) = 0.0406 *p* (1–3) = 0.0022
	abs	0.01 (0.01; 0.04)	0.01 (0.01; 0.03)	0.03 (0.02; 0.05)	*p* (1–3) = 0.0104 *p* (2–3) = 0.0022

Note: Data are presented as median (IQR). Kruskal–Wallis testing was used for analysis. ns—non significant differences (*p* > 0.05).

**Table 4:** Baseline characteristics of the patients included in the study.

Group	Age (Me, Q25–Q75)	Sex (%)	Fibrosis Stage (%)	ALT Levels (U/L, Me, Q25–Q75)	AST Levels (U/L, Me, Q25–Q75)	Bilirubin (mcmol/L, Me, Q25–Q75)
Chronic HBV	48, 36–66	29.4% male vs. 70.6% female	F0–F1—32.3% F2–F3—5% F4—55.8%	24, 18.5–37.6	29, 20.3–44.9	16.5 (12.2–29)
Chronic HCV	52, 39–67	41.6% male vs. 58.4% female	F0–F1—28% F2–F3—17% F4—11% 44% unspecified	57, 29.3–95.5	45, 30.3–93.3	32.5, 10–92.8
Healthy donors	40, 34–49	43% male vs. 57% female	-	-	-	-

## Data Availability

The data presented in this study are available on request from the corresponding author due to institutional policy.

## Abbreviations

The following abbreviations are used in this manuscript:

ALT	Alanine transaminase
AST	Aspartate transaminase
CM	Central memory
CTL	Cytotoxic T cells
EM	Effector memory
HBV	Hepatitis B virus
HCV	Hepatitis C virus
IQR	Interquartile range
Mcmol/L	Micromole per liter
Me	Median
Tc	Cytokine-producing cytotoxic T cells
TEMRA	Terminally differentiated effector memory T cells
Tfh	T follicular helper cells
Th	Helper T cells
U/L	Units per liter
WHO	World Health Organization
